# Modifying Hata-Davidson Propagation Model for Remote Sensing in Complex Environments Using a Multifactional Drone

**DOI:** 10.3390/s22051786

**Published:** 2022-02-24

**Authors:** Faris A. Almalki, Ben Othman Soufiene

**Affiliations:** 1 Department of Computer Engineering, College of Computers and Information Technology, Taif University, P.O. Box 11099, Taif 21944, Saudi Arabia; m.faris@tu.edu.sa; 2 PRINCE Laboratory Research, ISITcom, Hammam Sousse, University of Sousse, Sousse 4023, Tunisia

**Keywords:** IoT, propagation model, tuning models, remote sensing, drones, complex environment, thermal imaging, rescue operations

## Abstract

The coupling of drones and IoT is a major topics in academia and industry since it significantly contributes towards making human life safer and smarter. Using drones is seen as a robust approach for mobile remote sensing operations, such as search-and-rescue missions, due to their speed and efficiency, which could seriously affect victims’ chances of survival. This paper aims to modify the Hata-Davidson empirical propagation model based on RF drone measurement to conduct searches for missing persons in complex environments with rugged areas after manmade or natural disasters. A drone was coupled with a thermal FLIR lepton camera, a microcontroller, GPS, and weather station sensors. The proposed modified model utilized the least squares tuning algorithm to fit the data measured from the drone communication system. This enhanced the RF connectivity between the drone and the local authority, as well as leading to increased coverage footprint and, thus, the performance of wider search-and-rescue operations in a timely fashion using strip search patterns. The development of the proposed model considered both software simulation and hardware implementations. Since empirical propagation models are the most adjustable models, this study concludes with a comparison between the modified Hata-Davidson algorithm against other well-known modified empirical models for validation using root mean square error (RMSE). The experimental results show that the modified Hata-Davidson model outperforms the other empirical models, which in turn helps to identify missing persons and their locations using thermal imaging and a GPS sensor.

## 1. Introduction

The importance of having reliable wireless communication links and systems during or after manmade or natural disasters is highlighted in [1]. In 2017, Florida was hit by one of the most damaging hurricanes in American history, with winds reaching up to 220 km/h. This resulted in around 50% of cell sites going of service, as well as around 1.2 million subscribers losing their wireless connections. Therefore, during disaster scenarios, space-based wireless communication systems including satellites, aerial Platforms, and unmanned aerial vehicles (UAVs) could offer a wide range of services. These would include, for instance, evaluating damage zones, coordinating rescue teams activities, delivering first-aid kits, accounting for missing people, and delivering good-quality signals in disaster areas [2,3]. However, terrestrial communication links are often disrupted when disaster strikes, as they are more vulnerable to physical damage. Thus, outer-space communication systems would serve as better solutions [4,5].

**Figure 1:**
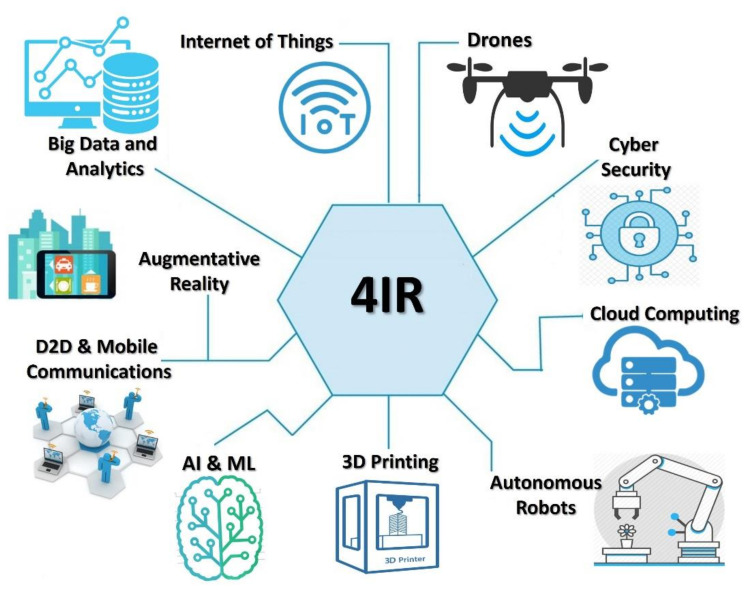
Technological pillars of the Fourth Industrial Revolution.

Unmanned aerial vehicles, including drones, and their wide applications are becoming one of the key elements in the current digital era, or Fourth Industrial Revolution (4IR), as Figure 1 shows. Coupling between 4IR pillars is seen as an approach that will become an integral part of society and even of our human bodies through such developments as smart cities, wearable technology, smart healthcare, smart farming, and new forms of artificial intelligence. Further, the integration between UAVs and the Internet of Things (IoT) would not only connect billions of items via the Internet, but also allow these connections to function automatically without human intervention. Cisco’s definition is “Bringing together people, processing, data and things to make network communications smarter and more valuable than ever, and transforming data into procedures that create new capabilities, richer experiences and an unprecedented economic opportunity for companies, individuals and countries”. Drones are increasingly integrated with evolving technologies, such as IoT, smart surveillance, artificial intelligence (AI), decision support systems, and machine learning (ML) techniques [6,7]. Therefore, drone technology has received researchers’ attention tin the context of remote sensing and rescue operations for various reasons. These reasons include drones’ reliability, flexibility, efficiency, applicability with line-of-sight (LoS) connectivity, low latency, and low manufacturing, launching, and maintenance costs.

This integration is vital, especially in the current digital era, when it is anticipated that over 50 billion devices will be connected to the internet by 2030, which in turn could generate real-time data that would open doors towards hyper-connected societies, and thus more global smart connectivity. Many applications could benefit from integration with UAVs, such as telecommunications, remote sensing, the monitoring of disaster-relief activities, high-resolution imaging, atmospheric studies, smart cities, smart agriculture, smart service delivery, wearable devices, traffic monitoring, border monitoring, news gathering, localization, and navigation. Hence, drones can provide seamless communication systems, as well as a variety of sensing applications and connections to grid networks, to support the idea of hyper-connected world in more efficient and timely manner [8,9].

To the best of our knowledge, this is the first work that considers a modified Hata-Davidson propagation model from a UAV perspective, despite UAVs’ advantages of accuracy, coverage, and correction factors. To obtain novelty and mark a noticeable shift from existing work, the key contributions of this work are as follows:

▪ Modifying the Hata-Davidson empirical propagation model based on RF drone measurement using the least squares tuning algorithm, which offers the advantages of accuracy, strong connectivity, and wide coverage.
▪ Building and testing a multifactional drone equipped with state-of-art 5G MIMO antennae as well as a thermal camera and sensors for search-and-rescue operations.
▪ Validating the results against other well-known modified empirical models in view of further development.

The rest of this paper is organized as follows. Section 2 presents related work, and then highlights this paper’s motivation. This is followed by Section 3, which describes the proposed model. Section 4 presents the implementation setup and discusses the results. Section 5 concludes the study.

## 2. Related Work

UAVs, including drones, are increasingly seen as innovative solutions for rapid search-and-rescue operations. They could provide many satellite advantages, but without the distance penalty. Receivers may experience a better signal quality as the system offers line-of-sight (LoS) communications and, hence, less propagation delay in relation to satellite systems [10,11]. Our review of relevant literature reveals that there has been some consideration of the capabilities of drones for emergencies, including different cameras, propagation models, altitudes, and search patterns. This section then concludes with a review of related research, and highlights this paper’s motivation.

The most critical requirement for search-and-rescue teams to be able to save people’s lives is having high-performance communication links. The authors of [11,12,13,14,15] focused on proposing different propagation models that could provide robust wireless communication between aerial stations and interdepartmental headquarters, including rescue teams. Hata and Okumura are typical empirical models, while air-to-ground and free-space are typical deterministic propagation models. These propagation models can measure path loss (PL) and coverage, which in turn are important parameters for monitoring wireless system performance and network planning. The results of using these propagation models in various emergency scenarios show that they exhibit advantages in relation to high altitude, wide coverage range, and adaption across different terrains. However, there are some shortcomings of existing models that should be addressed to enhance resilience and offer better quality of service (QoS) in harsh environmental conditions.

Utilizing a drone to detect humans and their location in real-time disastrous situations is simulated and experimented in [16]. The proposal aims to identify the location of survivors buried under wreckage using a camera module, a microcontroller, and a sensor. The results show that the drone with its payload can be a vital and cost-effective tool for detecting victims who are still alive in harsh environmental conditions. In [17], re-searchers introduced a drone prototype for intelligent search, rescue, and disaster recovery missions. A colored camera, a GPS sensor, machine learning, and image processing were the tools used to carry out the missions. The results show that the proposed prototype can operate autonomously and quickly for search operations.

A lifeguard drone flying at a 100 m altitude was designed to alert divers at beach locations and deliver an automatic inflating vest to drowning victims [18]. The initial call is started by a swimmer, who wears wristband, so they can press a button on the device; the drone can then the location using a GPS and ZigBee transmitter. The experimental results show a reasonable response from the drone. However, the use of a HD camera along with artificial intelligence are recommended to enhance the work.

The authors of [19] proposed an automated human detection system mounted on a drone, with components including RGB camera and GPS sensor, for search-and-rescue missions. The system aims to offer rapid localization and allocate a score of confidence for each detection. At a 15 m altitude, the results show moderate accuracy in detecting persons with a higher level of confidence. A fully autonomous drone equipped with human detection sensors, communication modules (e.g., GSM/GPRS module, GPS), and an object collision detection sensor for search-and-rescue operations is discussed in [20]. At a 7 m altitude, both simulation and trials have shown that using inexpensive and reliable sensors such as passive infrared (RIP) sensors, ultrasonic sensors, and Arduino microcontrollers offered the ability to detect humans trapped under ruins. However, the paper recommends thermal cameras and advanced sensors to enhance the accuracy and response times.

The authors of [21] present a drone that utilizes multi-sensors and geolocalization to detect survivors after natural disasters as part of search-and- rescue operations. The autonomous and robust drone is also equipped with an ultra-wideband radar, microphone array, camera, RFID reader, laser range finder and LIDAR at an altitude of 13 m. The results suggest the capability of early-stage survivor detection; however, the authors promise further development of the prototype in relation to the camera module and propagation model.

The authors of [22] used a drone as the first responder in natural and/or man-made disasters due to its rapid response, as well as its adaptability to particular environments. The first-responder drones (FRDs) system implemented a long-range (LoRa) system, which is enabled by low power-wide area network (LP-WAN), as well as a GPS module and a camera onboard. The results suggest that the proposed FRDs could respond within a range of 10–46 s of a footprint up to 30 km in rural areas; the range decreases as the drone moves towards urban areas. Moreover, increase the distance between a drone and the terrestrial receivers is an issue that is repeatedly highlighted in the literature. Where it is argued that optimizing propagation path loss and power consumption would be key parameters to enhance wireless connectivity at different drone altitudes.

In [23], a drone model is shown that uses a deep-learning algorithm to enhance aerial image processing for search-and-rescue operations that deal with human lives. At an altitude of 61 m, with a small onboard GPS device and a colored camera, the proposed drone taken thousands of human pictures, before using them as training, test, and validation primary data for the deep-learning algorithm. The results show a high level of sensitivity with an acceptable false-positive rate. For further work, thermal cameras at different drones altitudes could be used to improve the search-and-rescue operations further.

The authors of [24] present an architecture of human detection during or after natural disasters using UAVs integrated with head-mounted display (HMD) devices. This architecture aims to deliver a solution to protect human lives in search-and rescue operations. Support vector machines (SVM) and adaptive boosting (AdaBoost) are traditionally used for human detection via an RGB camera installed on an UAV. The experimental results show reasonable object detection architecture with search-and-rescue operations via RGB images. Thermal cameras are recommended to improve detection in low light and/or in camouflaging foliage scenarios.

In [25], a multipurpose UAV is presented to carry out a rescue operations due to avalanche events in mountains. The aerial platform is integrated with avionics that meet the environmental requirements for mountainous terrain (e.g., low temperatures, high altitude, and strong winds). The model uses a visible thermal camera for search-and-rescue missions involving missing persons in snow and in woods during any time of the day. Results show that the proposed model can operate autonomously and in quick search operations at different altitude. The thermal FLIR Tau 2 IR camera shows an acceptable level of performance for snow-covered bodies up to 20 cm. There is a trade-off between flight endurance and payload weight against power consumption.

The detection of the heat profile to locate missing persons using drones integrated with a thermal camera is considered in [26]. The proposal calculated target coordinates of a missing person using a closed-form formula. The results indicate that UAV can detect humans lying or sitting on the ground as well as identifying their location. The authors of [27] introduced an overview of drones used for hyperspectral remote sensing at low altitudes. The drone payload’s weight, size, flight duration and cost are criteria that help to choose the right payloads (e.g., sensors) and drone design.

The authors of [28] integrated a convolutional neural network (CNN) and an autonomous drone with a thermal camera to locate victims quickly and efficiently in disaster zones. The experimental results confirm that detecting victims from an aerial thermal view was achieved with AP = 82.49%. However, the drone’s altitude and coverage range were not clarified. Moreover, in [29], the CNN also used autonomous drone-surveillance and a RGB camera for search-and-rescue in natural disasters at 40 m altitude. The results confirm that detecting accuracy of victims from an aerial view was achieved with 98%. Further image processing and optimization techniques are introduced in [29,30], where a drone-based system is developed to detect the breathing movements of casualties or lost people within natural-disaster zones. The results show reasonable precision average measures from altitudes ranging from 2–8 m. Further work is recommended, involving tuning to high altitude and high image resolution, to gain better results.

The authors of [31] presented a multifunctional drone combined with a machine learning (ML) approach that helps in fighting against the coronavirus pandemic. The proposed drone, with its payload of thermal cameras and ML, can not only detect people wearing face masks in public for security reasons, but also help to sense elevated body temperatures in order to minimize cross-infection through close contact. Furthermore, the proposed work pays attention to the optimization of communication links for better wireless connectivity and power consumption. The authors of [32,33,34,35,36] covered different techniques that can allow UAVs to divide the target area for search and rescue operations. Where snooping or strips are the most well-known searching techniques.

In [37,38], the authors emphasize the importance of UAV trajectory design, which affects not only the communication and link budget between UAVs and ground users, but also UAVs’ energy consumption. They also considered a deep-reinforcement algorithm for trajectory design and power allocation optimization.

In the literature, a few types of camera have been used for detecting humans and/or wildlife animals from a bird’s-eye view on satellites or UAVs; they are mainly classified as thermal cameras and RGB cameras. However, this work focuses on using a thermal camera based on the heat signature of objects using mid- or long-wavelength infrared energy. It has the advantages of easy installation and usage, independent of light, the provision of fast and accurate measurements of objects, and limited privacy issues [39,40,41,42,43,44,45].

The authors of [46,47] studied a drone’s ad hoc network for mobility and service-oriented modeling using various protocols and techniques, such as neuro-fuzzy, and a quality of service provisioning framework for UAV-assisted environments. The results of these innovative approaches have emphasized the significant potential of enabling smart services.

Based on our review, our motivations and contributions are as follows. Our study aims to modify the Hata-Davidson empirical propagation model based on RF drone measurement using the least squares tuning algorithm. The optimum experimental configuration is considered to allow a drone with a thermal camera and other payloads flying over disaster zones in rugged areas to help identify lost people as part of robust and efficient search-and-rescue operations. The deployment of portable drones with thermal cameras and state-of-art 5G MIMO antennae, as well as sensors, for search-and-rescue operations is scarcely reported in the literature, let alone the optimization of a propagation model to ensure strong wireless connectivity and a wider coverage footprint. To the best of our knowledge, this is the first work to consider the Hata-Davidson propagation model from a UAV perspective, despite UAVs’ advantages of accuracy, coverage, and correction factors. To obtain novelty and mark a noticeable shift from existing work, six objectives were pursued:

- Investigate more empirical propagation models that suit the nature of low-altitude drones.
- Optimize an empirical propagation model that offers advantages (e.g., accuracy, suite different environment, strong received signal strength (RSS) connectivity, and wide coverage).
- Built a drone with onboard equipment (e.g., thermal FLIR lepton camera, 5G MIMO antenna, weather station, and GPS sensors).
- Identify the optimum wireless communication configuration of the drone (e.g., drone altitude, covered footprint, weather conditions (temperature, wind speed), longitude, and latitude). These parameters were linked to the optimized model to ensure cohesion and accurate results.
- Perform a hardware calibration before the implementation of the proposed design for testing and verification in a real scenario.
- Validate the results against other well-known modified empirical models for validation using RMSE.

## 3. Proposed Model and Experiment Setup

**Figure 2:**
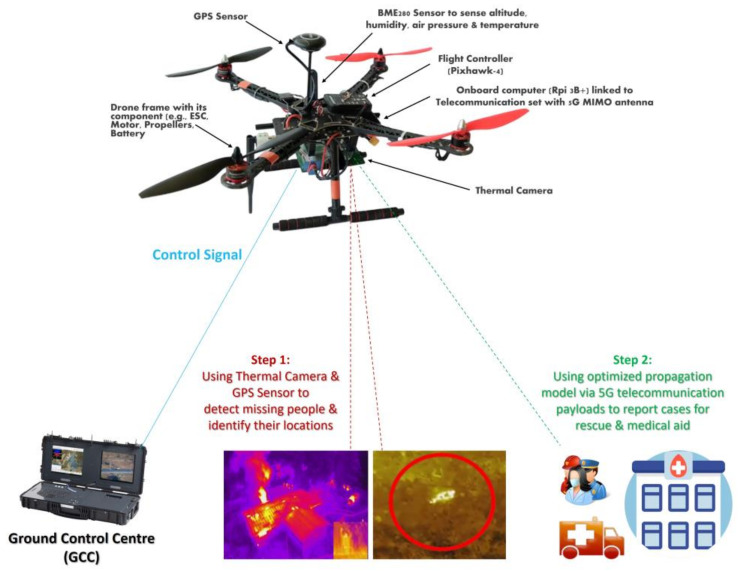
Proposed structure of a drone for use in search-and-rescue operations.

Emergency responders require real-time imagery and information in cases of disasters that threatens lives. These events involve large number of workers carrying out rescue missions for injured or missing people at rugged or isolated areas, which require more effort, cost, and risk. Figure 2 shows a proposed model architecture consisting of two main segments, a space segment, and a ground segment, to serve the purpose of carrying out search-and-rescue operations to detect casualties or lost people within rugged natural and/or manmade disaster zones using a drone integrated with 5G telecommunication payloads and a thermal camera. The space segment contains a drone with communication payloads such as a thermal camera, a GPS sensor, and telecommunication payloads with 5G MIMO antennae. The ground segment consists of fixed or mobile users, as well as a ground control center (GCC) that acts as the focal point between two segments and hosts gateways to external networks.

**Figure 3:**
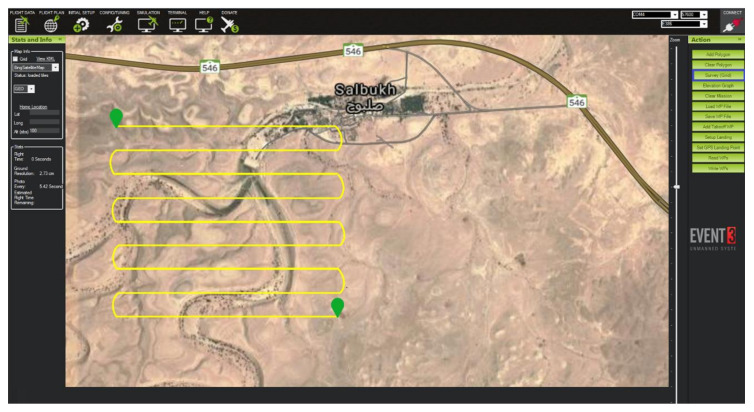
Strips searching technique performed by a drone.

In the literature, well-known search techniques that allow UAVs to divide a target area for search-and-rescue operations include strips, lines, or random techniques [34,36]. In our study, we used the strips technique, as Figure 3 shows, since it is the most suitable approach to achieving our research goal. To illustrate, when a drone conducts a search mission it makes sense to divide a target area into strips so that they can go over the divided areas one-by-one, performing only one turn at the end, which in turn achieves two goals. The first is coverage of each raw target area; the second is energy savings in comparison to other search techniques.

### 3.1. Conceptual Model

**Figure 4:**
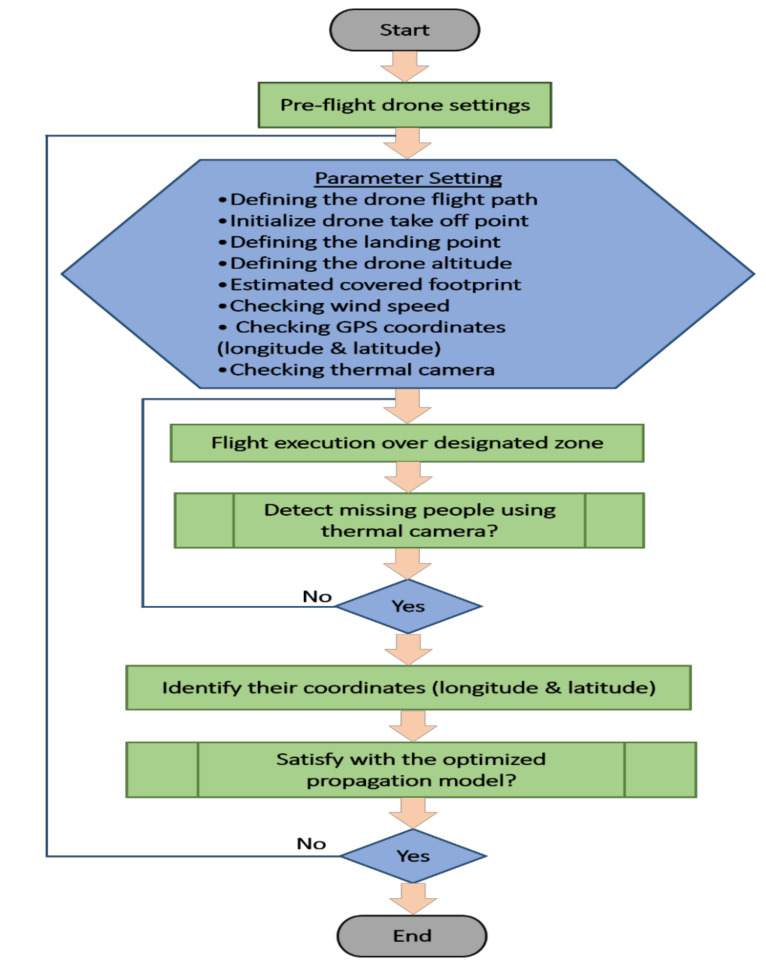
Flowchart of the proposed model and experiment setup.

Figure 3 displays the use of Mission Planner software, which is used as a ground control station for the drone, where vital configuration and setup can be performed to attain the optimum performance of aerial vehicles. The strips search technique is used when flying a drone to conduct search-and-rescue missions autonomously. When the drone flies autonomously to a targeted area, two main steps should be considered. The first step involves using thermal camera and a GPS sensor to detect missing people and identify their locations. The second step involves a reliance on the optimized Hata-Davidson propagation model, which enhances connectivity to report cases for rescue and medical aid. These two steps can be visualized in more detail in Figure 4, as a flowchart. The section includes two subsections. The first presents the mathematical concept supporting the proposed framework. The second describes the hardware and software architecture used in the experiment.

Notably, the experiment focuses on using a drone with a thermal camera and communication payloads to fly over disaster zones in rugged areas and detect lost people as part of robust and efficient search-and-rescue operations. Thus, an optimum-channel wireless communication model should be optimized to achieve four main objectives between the drone and the control station: 1—strong wireless connectivity; 2—wide coverage footprint; 3—high-throughput transmission; and 4—low power consumption and, thus, longer drone flight time.

### 3.2. Mathematical Calculation

This subsection aims to highlight the mathematical calculations for conducting the experiment from the space segment, which represents the drone, to the ground segment, which represents the GCC. This includes the Hata-Davidson empirical model [48,49], which is a radio propagation model used to predict path loss in fifth-generation multiple-input multiple-output (5G MIMO) antennae transmissions between drones and ground control stations (GCS). This is followed by the equations of the proposed optimization techniques for tunning the propagation model, called the “least squares tuning algorithm”. Additionally, more mathematical details are presented to calculate the related link budget parameters, such as: RSS, signal-to-interference-noise-ratio (SINR), and throughput (T). Additional to geolocation information using a GPS sensor that is used to identify the locations of casualties or lost people, and then notify authorities as to the target’s exact GPS coordinates. The Hata-Davidson empirical propagation model can be calculated as per (1) to (5) [48,49]:

(1) $$P_{L}\mathrm{HD}=P_{L}\mathrm{Hata}+A\;\left(h_{t},d\right)-S_{1}\left(d\right)-S_{2}\left(h_{t},d\right)-S_{3}\left(f\right)-S_{4}\left(f,d\right)$$

(2) $$d\;[\mathrm{km}]=2{\;E}_{r}[\cos^{-1}\left(\frac{E_{r}}{E_{r}+h_{t\;}}*\cos\left(\theta \right)\right)-\theta ]\;$$

For urban areas:

(3) $$a\left(h_{r}\right)=(3.2[\log\;\left(11.75{\;\times \;h}_{r}){]}^{2}\right)-4.9$$

For suburban, or rural areas:

(4) $$a\;\left(h_{r}\right)=\left(1.1\;\log\left(f\right)-0.7\right)\left(h_{r}\right)-\left(1.56\;\log\;\left(f\right)-0.8\right)$$

(5) $${P\;}_{L}\mathrm{Hata}=69.55+26.16\log\;\left(f\right)-13.82\;\log\;\left(h_{t}\right)-a\left(h_{r}\right)+\left[44.9-6.55\;\log\;\left(h_{t}\right)\right]\log\;\left(d\right)$$

where $P_{L}\mathrm{HD}$ denotes the path loss of Hata-Davidson, $h_{t}$ denotes the transmitter antenna altitude, $h_{r}$ denotes the receiver antenna altitude, $a\left(h_{r}\right)$ denotes the correction factor for the mobile antenna height, f denotes the carrier frequency, $E_{r}$ denotes the Earth radius, $P_{L}\mathrm{Hata}$ denotes the path loss of Hata, d denotes the distance at which the propagation model’s range is computed based on the elevation angle (θ), A and $S_{1}$ represent factors that extend the distance to 300 km, $S_{2}$ denotes the correction factor for the height $h_{t}$ of the base station antenna extending the value of $h_{t}$ to 2.5 km, and $S_{3}$ and $S_{4\;}$ represent the correction factors for the Hata-Davidson model that extend the frequency to 1.5 GHz [46,47].

To reach an optimum communication link with different environment characteristics, the least squares tuning algorithm is considered where tuning is necessary to adjust the Hata-Davidson propagation model’s parameters to the measured data. Mathematical equations for the least squares tuning algorithm are presented as per Equations (6) to (8).

(6) $$E=\sum_{i=1}^{n}{\left(Y_{i}-{\mathrm{PLHD}}_{i}\right)}^{2}$$

where the tuning algorithm is exemplified by the concept of minimizing the sum of the squares of the differences E between the measured data $Y_{i}$ and the predicted data ${\mathrm{PLHD}}_{i}$; n denotes the total number of used data. In the Hata-Davidson propagation model, we used two main tuning terms, as per Equations (7) and (8). These two terms reportedly affect propagation models, which are related to distance (d), and frequency (f).

(7) $$K_{1}=A\;\left(h_{t},d\right)$$

(8) $$K_{2}=\log\;\left(f\right)$$

where $K_{1}$ and $K_{2}$ denote the Hata-Davidson propagation model coefficients for the tuning and optimization process.

**Figure 5:**
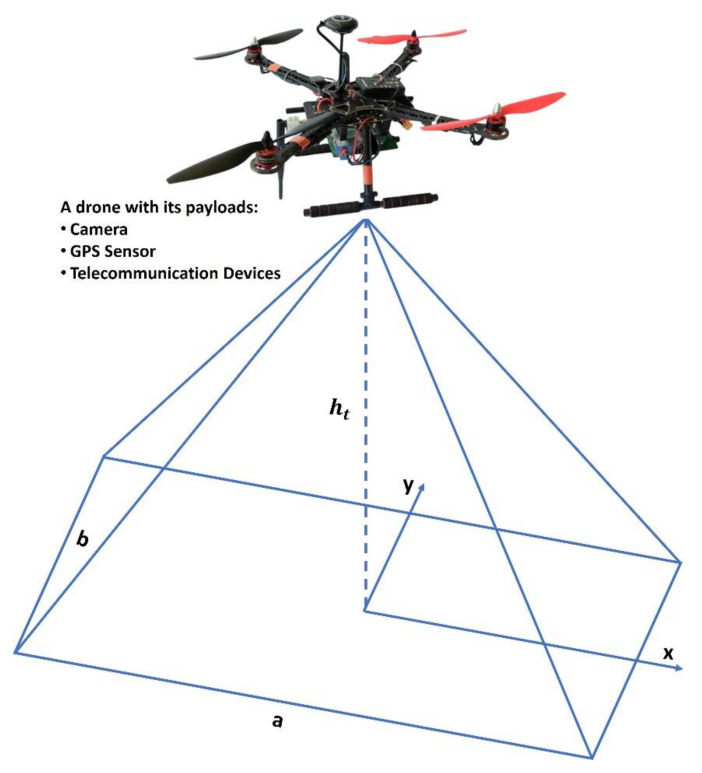
A drone with a bride’s eye view of the vertical coordinates of GPS positions.

The link budget parameters can be expressed by Equation (9) through to (11) [11]:

(9) $$\mathrm{RSS}=P_{t}+G\left(h_{t}\right)+G\left(h_{r}\right)-P_{L}\mathrm{HD}-L$$

(10) $$\mathrm{SNIR}=\frac{\mathrm{RSS}}{N+I}$$

(11) $$T=B\;\times \;\log\left(1+\mathrm{SNIR}\right)$$

where RSS denotes the received signal strength, $P_{t}$ denotes the transmitter power, $G\left(h_{t}\right)$ denotes the transmitter antenna height gain, $G\left(h_{t}\right)$ denotes the receiver antenna height gain, L denotes the connector and cable loss, N denotes the noise figure, I denotes the interference, B denotes the bandwidth, SINR denotes the signal-to-interference-ratio, and T denotes the throughput (b/s). The position of the drone is assumed to be at the centre of the image because the GPS module is placed above the camera. Therefore, the coverage of an image can be estimated using the camera’s field of view (FOV), as shown in Figure 5, while the target’s GPS coordinates can be calculated as per (12) to (19) [44]. Notably, the coverage of an image from an aerial perspective can be estimated using the camera’s field of view (FOV). This makes it possible to obtain an FOV with 180 degrees of peripheral vision. This is why it is presented as a rectangular shape.

(12) $$a=\frac{2h_{t}}{\cos\left(\frac{\mathrm{FOV}\;\left(x\right)}{2}\right)}$$

(13) $$b=\frac{2h_{t}}{\cos\;\left(\frac{\mathrm{FOV}\;\left(y\right)}{2}\right)}$$

(14) $$\mathrm{scale}\;\left(x\right)=\frac{a}{80}=\frac{2h_{t}}{80\left(\frac{\mathrm{FOV}\;\left(x\right)}{2}\right)}$$

(15) $$\mathrm{scale}\;\left(y\right)=\frac{b}{60}=\frac{2h_{t}}{60\left(\frac{\mathrm{FOV}\;\left(y\right)}{2}\right)}$$

where a denotes the length of the area, FOV (x) denotes the horizontal FOV of the camera, b denotes the width of the area, FOV (y) denotes the vertical FOV of the camera, scale (x) denotes the linear relationship between the x pixels and the distance, and scale (y) denotes the linear relationship between the y pixels and the distance. A target is assumed to be located on the (x, y) pixel in the photograph, and the offset of the target from the centre of the picture is as per (16):

(16) $$\mathrm{offset}\;\mathrm{target}=\left[\begin{array}{c} \mathrm{scale}\;\left(x\right)*x\\ \mathrm{scale}\;\left(y\right)*y \end{array}\right](m)$$

As the GPS location of the image frame is referenced from the centre of the image, the GPS must be corrected. In this way, missing persons on the edges of the frame can be located more accurately. The pixel values are given to the software in x and y Cartesian coordinates, then multiplied by the distance per pixel in their respective directions. The x and y directions are then rotated depending on the heading of the drone using the rotation of matrix, as per (17):

(17) $$R\begin{array}{c} c\\ w \end{array}=\left[\begin{array}{cc} \cos\left(\Psi \right) & -\sin\left(\Psi \right)\\ \sin\left(\Psi \right) & \;\cos\left(\Psi \right) \end{array}\right]$$

where ψ denotes the angle of the drone. Consequently, the position offset in the world frame can be solved as per (18):

(18) $$P=R\begin{array}{c} c\\ w \end{array}\mathrm{offset}\;\mathrm{target}=\left[\begin{array}{c} \mathrm{Px}\\ \mathrm{Py} \end{array}\right]$$

where P denotes the position offset in the world frame, Px denotes the latitude, and Py denotes the longitude.

(19) GPS target=GPS cam+PxfxPyfy

where the GPS target denotes the target’s GPS coordinates, and GPS cam denotes the longitude and latitude of the camera, fx and fy, respectively. Table 1 lists the abbreviations used in this paper.

## 4. Experiment Setup and Results

This section starts with information about the experiment’s setup and technical specifications. Next, a discussion of the experimental results, which starts by presenting the final configuration of the drone and the experiment location, is presented. This is followed by introducing the Hata-Davidson empirical propagation model with its tuned results, highlighting the results of the drone’s search-and-rescue missions using its components. When reaching the final setup and configuration of the drone with its components, all the components were calibrated and tested for both ground segments and space segments. Mission Planner, MATLAB, ThingSpeak, Blynk, and TCP/IP package were used as the software enablers for the communication, GPS coordination, and live tracking of the drone.

### 4.1. Experiment Setup and Technical Specifications

This subsection gives a brief outline of the experiment setup and the hardware specifications of its components. Table 2 highlights the configurations of the experiment’s components in both ground and space segments. A FLIR Lepton 2.5 thermal camera was used for aerial imaging to scan and search for lost persons in disaster zones. Raspberry Pi 3 B+ was used for controlling the thermal camera and GPS sensor of the universal asynchronous receiver transmitter (UART); these two components were linked synchronously to detect missing persons and identify their GPS coordinates. A BME280 sensor was used to sense the humidity, altitude, air pressure, and temperature measurements, since these are important for any aerial missions. A FOXEER 5.8 GHz Circular Polarized Omni Antenna is used. This circular, polarized, omnidirectional antenna can be used on both sides of the transmitters and receivers. A video transmitter and video receiver were used with wireless devices for transmitting and receiving the FLIR camera video.

The proposed drone used in this experiment features four propellers and few components. It has a frame that uses an F450 multicomputer quadcopter kit frame (PCB Version). An electronic speed controller (ESC) was used to control the speed of the motor and protect the batteries. Four propellers were used to provide lift to the drone by spinning to obtain different pressures between the top and bottom surfaces, which counteracts the force of gravity. Brushless DC motors were used and powered by a DC electric source via a switching power supply, which produced an AC electric signal to drive the motor. A drone battery was used due its advantages (e.g., light weight, high power, and quick charging). A flight controller (Pixhawk-4) was used to provide the standard for readily available, high-quality, and low-cost autopilot hardware designs. A transmitter remote controller was used to control the drone wirelessly; the signal/commands were then received by a radio receiver connected to a flight controller. An air receiver was used to receive commands from the transmitter to help to control the drone.

The rest of this subsection gives a briefed outline of the hardware specifications of the experiment’s components, which were:

FLIR Lepton 2.5 Thermal Camera used as a thermal sensor for imaging to scan and search for lost persons in disaster zones. The technical specifications are as follows. (50° HFOV, 60° diagonal, 80 (h) × 60 (v) active pixel. Thermal sensitivity <50 mK, Pixel Size: 17 micrometers. Frame rate: 9 Hz. Output format: User-selectable 14-bit, 8-bit, or 24-bit RGB. SPI video interfaces. Two-wire I2C-like serial-control interface. Fast time to image (<1.2 s). Low operating power, nominally 150 mW).

Raspberry Pi 3 B+ used for controlling the thermal camera and GPS sensor. The technical specifications are as follows. CPU: Quad-Core 64-bit 1.4 GHz. RAM: 1 GB LPDDR2 SDRAM. Wi-Fi: Dual-band 2.4 GHz + 5 GHz. Ethernet: 300 Mbps Gigabit. PoE HAT compatible. GPIO: Extended 40-pin header. Storage: Micro-SD card slot. Video: HDMI, DSI display port, CSI camera port, USB: 4 × USB 2.0 ports + Micro USB 5 V/2.5 A power.

GPS Module used to connect this module to the Raspberry Pi, controlled by a universal asynchronous receiver transmitter (UART). The technical specifications are as follows: Supply voltage: DC 3–5 V. Interface: UART (Serial). Data transfer rate: 9600 bits per second. Module size: 2.5 × 3.5 cm. Size of the ceramic antenna: 2.5 × 2.5 × 0.8 cm).

Drone Frame using an F450 multicomputer quadcopter kit frame (PCB Version). The technical specifications are as follows. Color: white/red. Wheel base: 17.7 in/450 mm. Weight: 248 g.

Electronic Speed Controllers (ESC) used to control the speed of the motor and protect the batteries. The technical specifications are as follows. Constant current: 30 A, compatible with LiPo battery. Input: 2–3 LiPo).

Brushless DC Motor used and powered by a DC electric source via a switching power supply, which produces an AC electric signal to drive the motor. The technical specifications are as follows. KV: 920. Weight: 53 g. ESC: 30 A.

Propellers used to provide lift to the drone by spinning to obtain different pressures between the top and bottom surfaces, which counteracts the force of gravity. The technical specifications are as follows. Ten-inch multi-copter RC quadcopter. Material: ABS. Size: 10 × 4.5’’. Diameter of shaft: 6.0 mm. Thickness of center: 9.0 mm. Recommended frame: 550–700 mm. Recommended motor: 800–1100 KV. Weight: 0.9 g.

Flight Controller (Pixhawk-4) used to provide the standard for readily available, high-quality, and low-cost autopilot hardware designs. The technical specifications are as follows. Processor: 32-bit, 168 MHz, 256 KB. Sensors: gyroscope, accelerometer. Power: 7 v. Interfaces serial ports. CAN, PWM or voltage input. External micro-USB port.

BME280 Sensor used to sense humidity, altitude, air pressure, and temperature measurements. The technical specifications are as follows. ESP32 to support IoT applications. Supply Voltage: 1.8–5 V DC. Interface: I2 C (up to 3.4 MHz), SPI (up to 10 MHz).

Transmitter Remote Controller used to control the drone wirelessly; the signal/commands are then received by a radio receiver connected to a flight controller. The technical specifications are as follows. RF range: 2.40–2.48 GHz. Bandwidth: 500 Hz. RF power: less than 20 dBm. Power: 12 V DC. Weight: 590 g.

Air Receiver used to receive commands from the transmitter that help to control the drone. The technical specifications are as follows. Channels: 6. Length: 36.6 mm. Width: 26.7 mm. Height: 12.7 mm. Weight: 0.9 g. Antenna length: 190 mm.

5G MIMO Antenna using FOXEER 5.8 GHz Circular Polarized Omni Antenna Cloud Spirit Tx Rx RP-SMA Male Black for the FPV multicopper. This circular, polarized omni-antenna can be used for both V$h_{t}$ antenna and V$h_{r}$ antenna. The technical specifications are as follows: Frequency range: 5.6–5.9 GHz. Gain: 3 DBi. Polarization: omni. Resistance: 50 Ω. Lightning protection: DC-grounded. Polarization: RHCP. Connector: RP-SMA. Size: 105 × 35 mm (feeder line 75 mm, weight: 12 g).

Video transmitter and video receiver using wireless devices for transmitting and receiving the FLIR camera video. The technical specifications are as follows. Transmitter antenna gain: 2 dB. Frequency: 5.8 G. Transmitting power: 600 Ma. Weight: 85 g. Receiver antenna gain: 2 dB. Power input: 12 V. Weight: 85 g.

### 4.2. Experiment Results and Discussions

**Figure 6:**
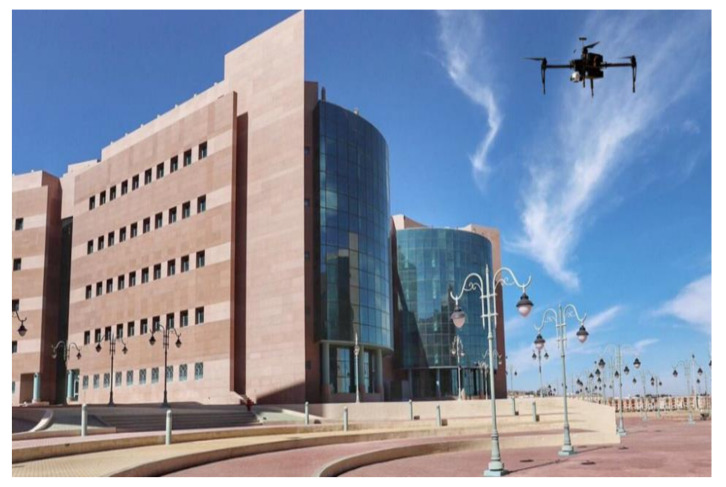
The proposed drone in the sky above Taif university campus for search-and-rescue trial.

Figure 6 illustrates the drone in the sky for search-and-rescue operations. The trial took place at the new campus of Taif university on 20 February 2021 at 1 p.m., at an altitude of 15 m. While the campus longitude is 40.4867323, and the latitude is 21.3320348. The reason why the trail took place on Taif university campus is that we obtained permission from the campus administration to fly drones at designated sites following the rules outlined by the university. Additionally, the ability to fly a drone over a limited number of people during lockdown was another reason to consider the campus as low-risk. Further reasons included accessibility to laboratories and hardware material, as well as the possibility to include some professional volunteers acting as missing people under objects such as trees or buildings.

**Figure 7:**
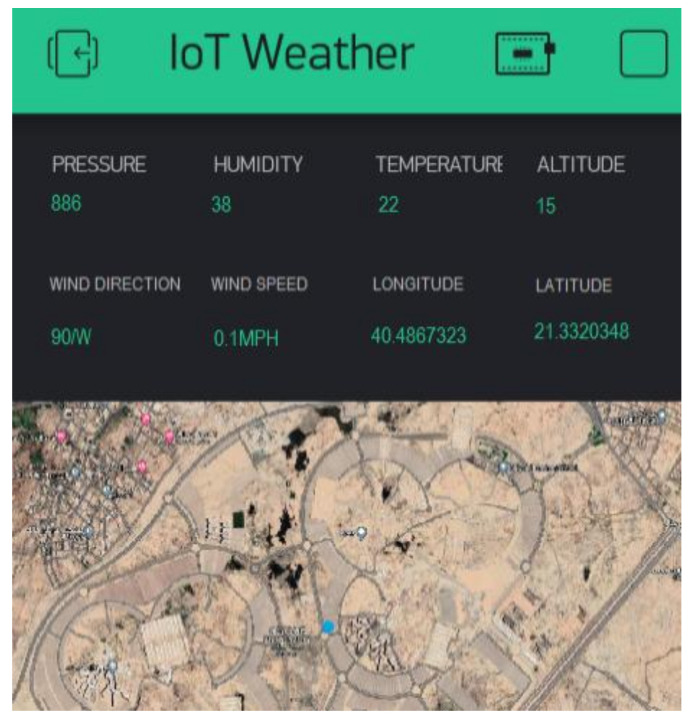
Weather conditions dashboard using Blynk application.

Figure 7 shows the weather conditions dashboard using the Blynk software application integrating the BME280 weather station sensor with the NEO6M GPS sensor, which were turn these connected to the Raspberry Pi 3 B+. Notably, Blynk is the most popular IoT interface platform for connecting any portable devices (e.g., laptop, mobile phone, tablet, smart watch) to the cloud. It is well known for allowing users to design apps to control their IoT devices, analyze telemetry data, and manage deployed products at scale. It can also support both Android and IOS operating systems. Five weather measurements besides altitude, longitude, and latitude were considered: temperature, pressure, humidity, wind direction, and wind speed. These parameters were considered due to their importance in determining the optimum flight configuration.

The RSS parameter is useful when calculating path loss (PL) since it is related to the distance between the transmitter and the receiver through an adequate propagation model. Although RSS calculations are quite simple to perform, achieving an optimized propagation model might be the opposite. Therefore, optimizing propagation models is crucial for the IoT, as it is the key foundation for communication resource allocation, localization, interference management, and sensing. Propagation path loss is a useful approach for monitoring system performance, network planning, and coverage to achieve perfect reception. Thus, the proposed modified Hata-Davidson empirical model is a noticeable contribution of this paper for calculating path loss for two reasons. First, the consideration of the elevation angle in relation θ to calculate the distance d that suits the drone’s nature as a space-based system. Second, the enhancement of the Hata-Davidson empirical model’s outcomes using experimental work with the least squares tuning algorithm. This adaptation yields improved coverage footprint, LoS connectivity, and RSS results from the receiver side.

**Figure 8:**
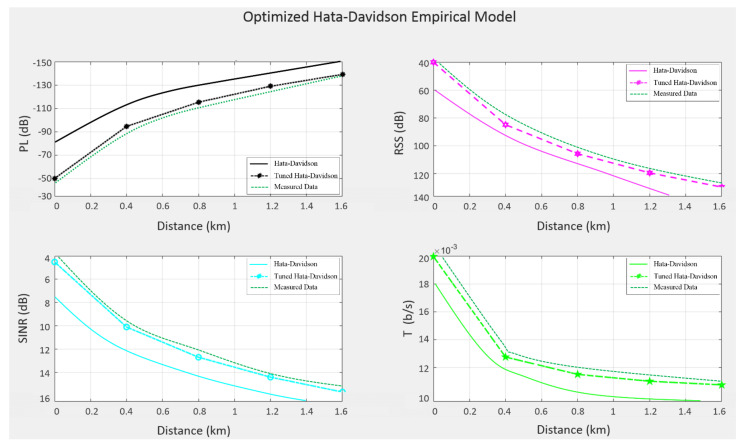
GUI of link budget plots of modified Hata-Davidson empirical model at 15 m altitude.

Figure 8 shows a GUI of the link budget plots of PL and the rest of the link budget parameters at an altitude of 15 m. Furthermore, it shows the Hata-Davidson model, modified-Hata-Davidson model, and measured data integrated between the communication payload on the drone and the receiver that connected to MATLAB. Notably, a MATLAB toolbox was used for predicting the link budget parameters. As can be seen, the PL increased with distance, while the RSS decreased as the PL increased. The average PL ranged between −150 dB and −50 dB, while the RSS ranged between 40 dB and 140 dB. Noticeably, the RSS was improved when increasing the transmitter altitude or transmission power. The SINR produced reasonable results: it increased as the transmitter altitude increased, due to the increase in coverage. However, T decreased with altitude, high PL, and shadowing. In general, the optimized Hata-Davidson model showed improvement in comparison to the non-optimized model in terms of connectivity, footprint, and RSS, which in turn led to the enhancement of communication links between the drone on one hand, and the GCS and rescue teams on the other hand. Notably, 15 m was found to be the optimum drone altitude based on the characteristics of the experimental area. Therefore, the altitude parameter can be changed based on the features of the covered area.

Unsurprisingly, the modified Hata-Davidson model displayed a good-level of improvement when using the proposed algorithm of least squares tuning. Another observation is that the measured data showed similar characteristics using modified Hata-Davidson model, which is a good performance indicator of the proposed least squares tuning algorithm. More details are presented on Table 3, which shows the two Hata-Davidson model coefficients before and after the tuning process. Once again, the table emphasizes the performance of the modified Hata-Davidson model against the non-modified model.

**Figure 9:**
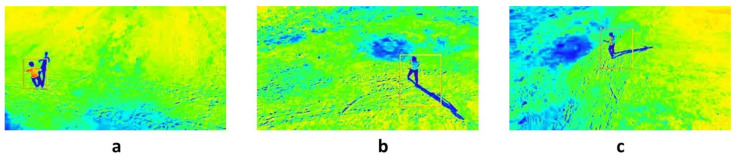
Vertical view using thermal camera to detect person at different altitudes: (**a**) 5 m, (**b**) 10 m, (**c**) 15 m.

**Figure 10:**
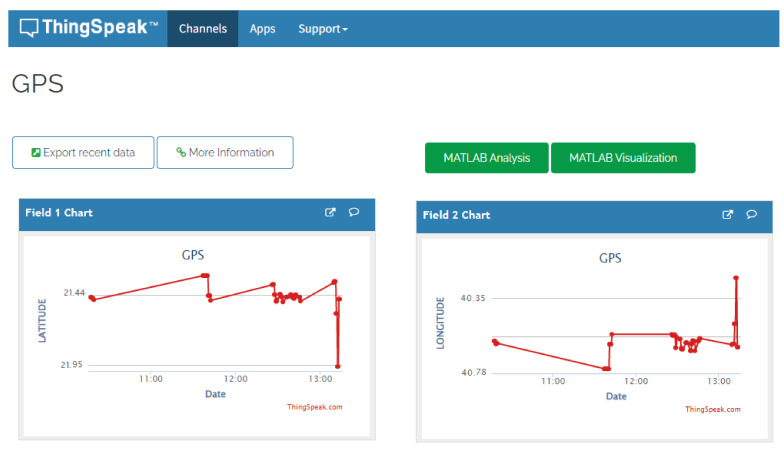
GUI channels of latitude (**left**), and longitude (**right**) from GPS sensor with satellite map view.

At the 15 m drone altitude determined previously, the drone was able to send the video from the FLIR lepton camera using the TCP/IP protocol. This TCP/IP protocol package was used to perform basic wireless network communication protocol between the drone and the ground station, as well as locating the positions of missing persons using the GPS sensor. The results of the vertical view using t the thermal camera to detect persons at different altitudes are shown on Figure 9. The figure shows a person labeled with a red circle from heights of 5, 10, and 15 m, respectively. Clearly, reasonable thermal visuals were observed from these altitudes; however, as the altitude increased above 15 m both low image quality and transmission delay became noticeable. Despite the high cost, a powerful thermal camera would enhance the thermal imaging quality of these results. Figure 10 demonstrates how the ThingSpeak website was used to provide channels for MATLAB users to help convert the NMEA data of the GPS module into an understandable format, before it was stored and presented as a satellite view on a specific web page. Further, these channels in ThingSpeak stored the latitude and longitude values, which helped track the drone’s movement and live location during the trial at Taif university. The figure shows the GUI of the longitude and latitude from the GPS sensor, along with a satellite map view of the detected person using the thermal camera. These results show the applicability of coupling a drone with IoT devices to carrying out intelligent search, rescue, and disaster-recovery missions.

To validate the proposed modified Hata-Davidson model against other well-known propagation models, the root mean square error (RMSE) is used. This study concludes with a comparison between the modified Hata-Davidson model and the SUI, Cost-231 Hata, and Okumura empirical propagation models for validation, presented in Table 4 [48,49]. The propagation models were selected as representatives of their empirical types along with their main parameters of coverage distance, transmitter antenna heights, receiver antenna heights, and high range of frequency bands. Additionally, these models exhibit better performance across different terrains in relation to other models. Furthermore, the Hata-Davidson model has the advantage of featuring some correction factors that yield high prediction accuracy. The complexity of the Hata-Davidson model is its only downside.

The RMSE is a quality estimator derived from the square of Euclidean distance, where it is always a positive value, with the error reducing as the error reaches zero. The RMSEs for all the considered propagation models are shown between the measured data and the predicted path loss, which in turn affects the rest of the link budget parameters. Clearly, the modified Hata-Davidson model performed the best compared to other models, since it had the lowest RMSE, of 3 dB.

## 5. Conclusions

Reliable wireless communication links and systems are vital during natural disasters and/or man-made disasters, which can be a matter of life and death. Thus, drones could offer a wide range of services in a robust and cost-effective manner, including evaluating damage zones, coordinating rescue teams’ activities, saving people’s lives, accounting for missing people, and delivering good-quality signals in disaster areas. This paper has presented both software and hardware work to build a drone equipped with a thermal camera besides its communication payload to fly over disastrous zones to help identify lost people as part of robust and efficient search-and-rescue operations.

The proposed modified Hata-Davidson empirical propagation model is introduced using the least squares tuning algorithm, and it shows outstanding performance in comparison to other empirical models using RME for validation. Moreover, the modified Hata-Davidson model is a noticeable contribution for calculating the path loss not only due to its consideration of the elevation angle in relation θ to calculate the distance d that suits the drone’s nature as a space-based system, but also to its enhancement of the Hata-Davidson model’s outcomes using the least squares tuning algorithm. This adaptation yields improved coverage footprint, LoS connectivity, and RSS results from the receiver side.

The Hata-Davidson model propagation model was selected as a representative of its empirical type, along with its main parameters of coverage distance, transmitter antenna height, receiver antenna height, and high range of frequency bands. The model exhibits better performance across different terrains, as well as some correction factors that yield high prediction accuracy in relation to other models. The complexity of Hata-Davidson model is its only downside.

The experimental results show that up to 15 m of altitude, the drone with its current capabilities was able to identify a missing person via thermal imaging, besides detecting their GPS location. Therefore, this work can help governments, emergency centers, or civil defense organizations to carry out search-and rescue-operations in a timely and efficient fashion, especially when accessing rugged areas and finding missing persons. To provide more details about the proposed model’s advantages in relation to existing models, Table 5 is presented.

Further work can be elaborated in four possible directions to enhance these results. 1—Despite the high cost, a more powerful thermal camera would enhance thermal imaging quality. 2—Using a radio frequency identification (RFID) sensor, which can detect the signals of electronic devices, would offer the further element of detecting lost persons who do not appear on thermal camera images. 3—Using solar panels on larger drones would enhance the flying time. 4—Considering high-frequency bands, such as 12 GHz or higher.

## Figures and Tables

**Table 1:** List of abbreviations.

Abbreviations	Description	Abbreviations	Description
$P_{L}\mathrm{HD}$	Path loss of Hata-Davidson	$G\left(h_{t}\right)$	Transmitter antenna height gain
$P_{L}\mathrm{Hata}$	Path loss of Hata	$G\left(h_{r}\right)$	Receiver antenna height gain
$A\;(h_{t},d)$	Correction factors for extend distance and transmitter antenna altitude	L	Connector and cable loss
$S_{1}\left(d\right)$	Correction factors for extend distance	SNIR	Signal-to-interference-ratio
$S_{2}\left(h_{t},d\right)$	Correction factor for transmitter antenna altitude	N	Noise figure
$S_{3}\left(f\right)$ $,\;S_{4}\left(f,d\right)$	Correction factors that extend frequency	I	Interference
θ	Elevation angle	T	Throughput
d	Distance of the propagation model’s range	B	Bandwidth
f	Carrier Frequency	a	Length of GPS area
$E_{r}$	Earth radius	FOV (x)	Horizontal field of view of the camera
$h_{t}$	Transmitter antenna altitude	B	Width of GPS area
$h_{r}$	Receiver antenna altitude	FOV (y)	Vertical field of view of the camera
$a\left(h_{r}\right)$	Correction Factor for mobile antenna height	Scale (x)	Linear relationship between x pixels and distance
E	Error function	Scale (y)	Linear relationship between y pixels and distance
$Y_{i}$	Measured data	$R\begin{array}{c} c\\ w \end{array}$	Rotation matrix
${\mathrm{PLHD}}_{i}$	Predicted data	ψ	Angle of drone
$K_{1}$	Hata-Davidson model coefficient 1	P	Position offset in the world frame
$K_{2}$	Hata-Davidson model coefficient 2	Px	Position latitude
RSS	Received Signal Strength	Py	Position longitude
$P_{t}$	Transmitter power	fx	GPS cam longitude
RMSE	Root Mean Square Error	fy	GPS cam latitude

**Table 2:** The configurations of the experiment’s components.

Thermal camera and GPS connection with Raspberry Pi 3 B+	** 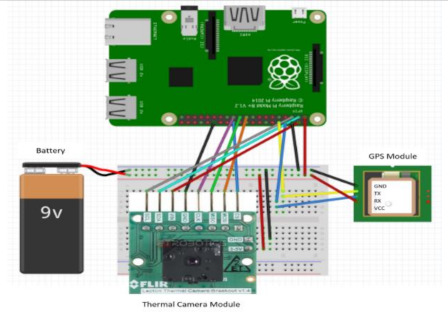 **
BME280 weather sensor connection with Raspberry Pi 3 B+	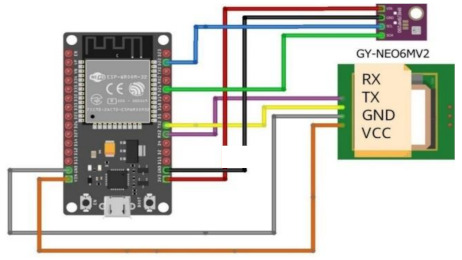
Plug Wi-Fi antenna in Raspberry Pi USB port	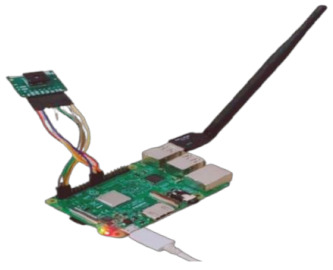
Drone components	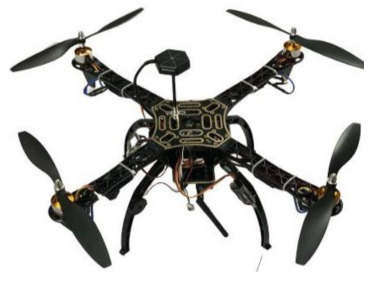
Connect the Pixhawk to a computer and mission planner software	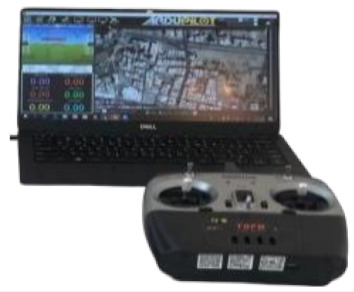

**Table 3:** Two Hata-Davidson model coefficients.

Hata-Davidson Model Coefficients	Before Least Squares Tuning Algorithm	After Least Squares Tuning Algorithm
$K_{1}$	10.41	6.53
$K_{2}$	13.75	8.92

**Table 4:** Validation results of the modified Hata-Davidson model against other propagation models.

Model	PL (dB)	RSS (dB)	SINR (dB)	T (mb/S)	Distance (km)	RMSE (dB)
SUI	−158.49	148.49	17.89	8.77	1.5	11
Cost-231 Hata	−161.35	151.35	19.02	9.24	1.6	12
Okumura	−159.48	149.48	17.63	8.65	1.5	9
Hata-Davidson	−150.72	140.72	15.79	10.19	1.3	7
Tuned Hata-Davidson	−135.3	125.3	14.68	11.43	1.6	3

**Table 5:** Comparison of proposed model against existing models.

Ref.	Altitude	Propagation Model	Frequency	Antenna Type	Human Detecting	Weather Station	GPS	Thermal Camera	Image Capturing
[16]	NA	Free-space	NA	Omni	√	×	√	×	√
[17]	100 m	Free-space	915 MHz	Directional	√	×	√	×	×
[18]	15 m	Free-space	2.4 GHz	Omni	√	×	√	×	√
[19]	7 m	Free-space	1.8 GHz	Omni	√	×	√	×	×
[20]	13 m	Free-space	2.4 GHz	Omni	√	×	×	×	√
[21]	NA	Free-space	863 MHz	Directional	×	×	√	×	×
[22]	61 m	Free-space	NA	Directional	√	×	√	×	√
[23]	NA	Free-space	NA	Directional	√	×	√	×	√
[24]	5 m	Free-space	457 kHz	Directional	×	×	√	√	√
[25]	50 m	Free-space	1.4 GHz	MIMO	√	×	√	√	√
[26]	NA	Free-space	NA	Omni	√	×	×	√	√
[27]	40 m	Okumura	NA	Directional	√	×	×	×	√
[28]	5 m	Free-space	NA	Omni	√	×	√	×	√
[29]	4–8 m	Free-space	NA	Omni	√	×	√	×	√
[30]	21 m	Two-rays	3.5 GHz	Omni	×	×	√	√	√
Proposed Model	15 m	Optimized Hata Davidson	5.8 GHz	5G MIMO	√	√	√	√	√

√ Refers to an element is existed. × Refers to an element is not existed.

## Data Availability

The data used to support the findings of this study are included within the article.
